# High-resolution grids of daily air temperature for Peru - the new PISCOt v1.2 dataset

**DOI:** 10.1038/s41597-023-02777-w

**Published:** 2023-12-01

**Authors:** Adrian Huerta, Cesar Aybar, Noemi Imfeld, Kris Correa, Oscar Felipe-Obando, Pedro Rau, Fabian Drenkhan, Waldo Lavado-Casimiro

**Affiliations:** 1 Servicio Nacional de Meteorología e Hidrología (SENAMHI), Lima, Perú; 2 Departamento de Física y Meteorología, Universidad Nacional Agraria La Molina (UNALM), Lima, Perú; 3 Image Processing Laboratory, University of Valencia, 46980 Valencia, Spain; 4 High Mountain Ecosystem Research Group, National University of San Marcos, 15081 Lima, Peru; 5 Institute of Geography, University of Bern, Bern, Switzerland; 6 Oeschger Centre for Climate Change Research, University of Bern, Bern, Switzerland; 7 Centro de Investigación y Tecnología del Agua (CITA), Departamento de Ingeniería Ambiental, Universidad de Ingeniería y Tecnología (UTEC), Lima, Perú; 8 Geography and the Environment, Department of Humanities, Pontificia Universidad Católica del Perú, Lima, Peru; 9 Present Address: Institute of Geography and Oeschger Centre for Climate Change Research, University of Bern, Bern, Switzerland

**Keywords:** Atmospheric science, Climate-change impacts

## Abstract

Gridded high-resolution climate datasets are increasingly important for a wide range of modelling applications. Here we present PISCOt (v1.2), a novel high spatial resolution (0.01°) dataset of daily air temperature for entire Peru (1981–2020). The dataset development involves four main steps: (i) quality control; (ii) gap-filling; (iii) homogenisation of weather stations, and (iv) spatial interpolation using additional data, a revised calculation sequence and an enhanced version control. This improved methodological framework enables capturing complex spatial variability of maximum and minimum air temperature at a more accurate scale compared to other existing datasets (e.g. PISCOt v1.1, ERA5-Land, TerraClimate, CHIRTS). PISCOt performs well with mean absolute errors of 1.4 °C and 1.2 °C for maximum and minimum air temperature, respectively. For the first time, PISCOt v1.2 adequately captures complex climatology at high spatiotemporal resolution and therefore provides a substantial improvement for numerous applications at local-regional level. This is particularly useful in view of data scarcity and urgently needed model-based decision making for climate change, water balance and ecosystem assessment studies in Peru.

## Background & Summary

Air temperature is a fundamental parameter of the climate system, which is required for various applications such as ecology^1^, hydrology^2^, public health^3^, agriculture^4^, climate variability, and climate change^5,6^. Typically, temperature values are obtained from meteorological stations and show high accuracy and temporal resolution but do not capture information for an entire unit or region of analysis. Therefore, gridded global- or continental-scale databases, derived from interpolated^7^, reanalyzed^8^ and/or combined^9^ *in-situ* and surface remote sensing data, are widely used. While each dataset offers several advantages for specific applications, limitations related to complex topography, spatial resolution, and the amount of assimilated data reduce their reliability^10,11^. In recent years, gridded high-resolution climate datasets at national and sub-national scales have been produced to close this gap^12–18^.

A broad range of methods exists for creating gridded air temperature data based on weather stations. Traditionally, they have been divided into geostatistical, non-geostatistical, and combined methods^19,20^. Although these methods are widely used and provide high efficiency, more recent procedures based on artificial intelligence including deep learning^21,22^ and machine learning^23,24^ are gaining relevance due to their ability to work with large amounts of data and capture non-linear and multivariate relationships^25^. However, the reduced capacity to estimate the value outside the range of the training data limits its use in large regions with low station density^26,27^. Besides, since the relationship between air temperature and auxiliary spatial predictors varies on spatiotemporal scales, recent research has also highlighted the importance of non-stationarity in the spatiotemporal domain by building local models in contrast to global estimation models^13,28–32^. The diversity of methods has advantages and disadvantages regarding data availability, computational efficiency, computational cost, and estimation accuracy. Therefore, the method selected must be suitable or at least adapted to the purpose and study area.

In South America, only few efforts have been undertaken to create gridded temperature datasets, mainly because of the low density of weather stations or the lack of long-term data series. However, there are significant advances in the construction of gridded datasets in countries such as Brazil^33,34^, Chile^35^, and Bolivia^36,37^. For Peru, only two databases exist currently. The first is a gridded monthly-scale product for 1964–2014 at 5 km spatial resolution (henceforth “VS2018”) developed by Vicente-Serrano^38^. The second is a gridded daily-scale product for 1981–2016 at 10 km developed by the National Service of Meteorology and Hydrology (SENAMHI). SENAMHI introduced this product as part of the Peruvian interpolated data of the Climatological and Hydrological Observations of SENAMHI (PISCO), denominated PISCOt v1.1^39^. Since its release, PISCOt has been applied in numerous areas of research and operation^3,40–45^. Due to the increasing availability of observed data and the need for higher spatial resolution, it is crucial to account for gridded air temperature datasets that allow modelling and process understanding at local scales, e.g., at the catchment level. Previously applied techniques^46–49^, show that such a product can be optimised by enhancing the temporal homogeneity of the observed data and also by using topographic and climatic co-variables. Among the applied remote sensing data, Land Surface Temperature (LST) is the most frequently used parameter because it improves both the numerical accuracy and the spatiotemporal details of the interpolated air temperature.

Here, we present an updated version (v1.2) of PISCOt that consists of a gridded daily dataset for maximum (Tmax) and minimum (Tmin) air temperature at a spatial resolution of 0.01° (≈1 km) for the period 1981–2020. The updated version of PISCOt is essential for two main reasons: (i) it provides high-resolution estimates of daily Tmax and Tmin in a data scarce region taking into account steep climatic gradients that occur over complex terrain; and (ii) it provides the basis for further applications such as studies related to climate change analysis, hydrological modelling, and ecology, among others.

## Methods

### Workflow for generation of the data

Missing, inhomogeneous, and non-quality-controlled data are a typical concern in hydro-climatological studies. Particularly in regions with low financial resources and limited technical and institutional capacities, weather station networks are often sparse with poor coverage in rural and remote areas, many stations do not work appropriately, and quality control systems are inefficient^50,51^. In Peru, quality issues with station data are especially challenging due to the complex topography leading to steep climatic gradients^52,53^. The development of PISCOt requires therefore careful selection and pre-processing of the station observations before spatial interpolation can be applied.

The selection of the horizontal resolution is crucial in the spatial interpolation process. From a climatological perspective, deriving coarser products rather than topoclimatic-scale products^13^ (kilometer or sub-kilometer) based on sparse interpolated observations does not yield additional information^54^. The underlying station distribution mostly defines the effective resolution, and it can be different from the target grid spacing^55–57^. However, from the user’s perspective, higher-resolution data can be more desirable since they are urgently needed for practical applications^58^. This is because these applications require a clear characterization of local gradients which in complex terrain might occur over shorter distances. An interpolation approach of air temperature based on high-resolution spatial predictors (0.01°≈1 km) is advantageous, especially in extremely complex mountain terrain such as the Andes, to properly account for the orographic gradients in a wide range of applications. Additionally, using high-resolution data makes it easier to interpret satellite observations or use them in hydrological models without further downscaling.

**Fig. 1:**
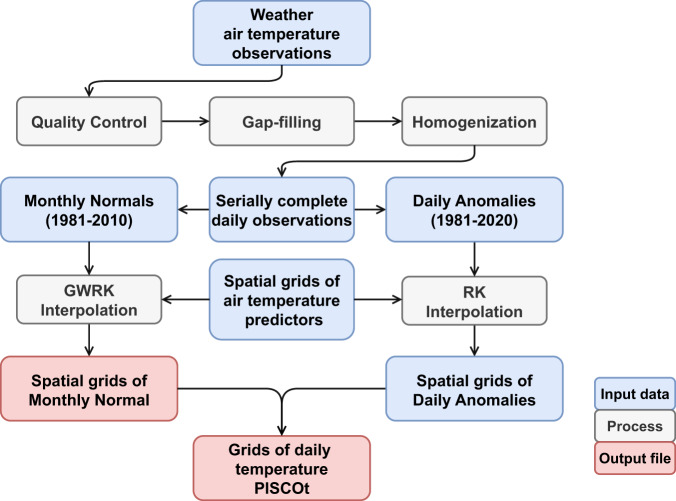
Schematic overview of the development of the daily air temperature gridded dataset (PISCOt). Input data, related processes, and main output files are specified. Spatial interpolation uses the Regression Kriging (RK) and Geographically Weighted Regression Kriging (GWRK) techniques.

Therefore, the workflow for the development of the new PISCOt dataset includes four steps: (i) quality control, (ii) gap-filling, (iii) homogenisation of weather stations, and iv) spatial interpolation (Fig. 1). In step (i), statistical and visual techniques were applied to remove erroneous data in the times series of Tmax and Tmin. For (ii), all time series were gap-filled using data from neighbouring stations. The previously gap-filled data were then homogenised in step (iii) to reduce temporal inhomogeneities. Once a complete and homogenised database of Tmax and Tmin observations was established, we proceeded to step (iv). A climatologically based interpolation approach^59–62^ was used, where the spatial interpolation was divided into the mean monthly normal and anomalies, and then aggregated to obtain the final product. Topographic and remote sensing data served as a basis to estimate air temperature at the country scale. The following sections provide the data sources and four development steps in more detail.

### Weather station data

#### Data source

**Fig. 2:**
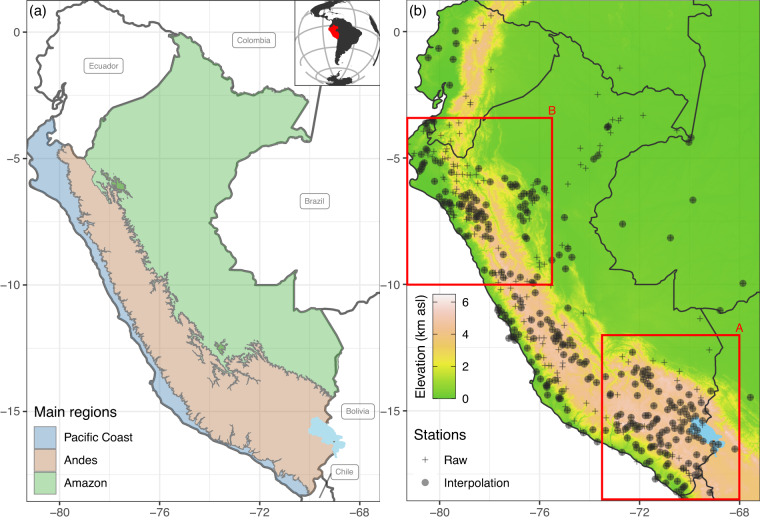
(**a**) Study area of Peru and its three main regions: Pacific Coast, Andes, and Amazon. The panel in the upper right corner shows the location of the study area in South America. (**b**) Spatial distribution of 462 available time series (Raw) for daily maximum (Tmax) and minimum (Tmin) temperature. After the data pre-processing, 302 time series were used for spatial interpolation (Interpolation). The red boxes represent the southern (A) and northern (B) Andes areas of Peru. Lake Titicaca is shown in light blue.

The database used in this study belongs to the Peruvian weather service (SENAMHI, https://www.senamhi.gob.pe) and includes 430 daily series of Tmax and Tmin (Fig. 2). To obtain a better spatial representation of the country boundaries (Fig. 2a), data from adjacent countries were used, such as the Ecuadorian Institute of Meteorology and Hydrology (INAMHI, https://www.inamhi.gob.ec), the Colombian Institute of Hydrology, Meteorology and Environmental Studies (IDEAM, http://www.ideam.gov.co), the Brazilian Institute of Meteorology (INMET, https://portal.inmet.gov.br) and the Climate Explorer portal of the Chilean Center for Climate and Resilience Research (CR2, https://explorador.cr2.cl). Consequently, we obtained a large set of climate data from Ecuador (18 time series), Colombia (3), Brazil (5), Bolivia (3), and Chile (3), representing a total of 462 potential time series (Fig. 2b). Due to restrictions on South American meteorological services, the raw data from the weather stations cannot be distributed with this publication. Readers that wish to obtain the primary data should apply to contact each agency or institution previously mentioned. It is important to note that while a substantial portion of the raw data is openly accessible, several data series remain restricted and can only be accessed upon request. Researchers are referred to revise the data provided by each institution via their official webpage and for further data requests contact each agency or institution individually.

The spatial distribution of the stations is highly uneven in the study area. While in the Amazon region, only a limited number of stations exists, station density in the Andes is higher and largest at the Pacific Coast (Fig. 2). Depending on the altitude, there was a lower (higher) density of stations between 1000 and 2000 masl (0–1000 masl and >3000 masl)^63^. Thus, the spatial distance between stations varied considerably. The earliest observations started in the 1930s, with a significant increase up to date. Due to political instability and social conflicts (Supplementary Fig. 1), two episodes of under-reporting occurred before 1960 and during the 1980s. Due to the low reliability of data before the 1980s, the gridded product only covers the period 1981 to 2020. In addition, only stations with at least five years of data (365 days of the year repeated at least five times) were used. The 5-year threshold was chosen based on the finding that at least 5–7 years of observations are required before pairwise relationships between stations stabilise^13,64,65^.

#### Quality control

The quality control (QC) of the air temperature series comprised the following steps:

1. Obvious errors: conversion of numerical values (−999, −99.9, −88.8) to empty values, and removal of duplicate or incorrectly formatted dates.
2. Extreme values: flagging of daily extreme (low and high) air temperature values based on physical and statistical values. The physical maximum and minimum limits for Tmax (Tmin) were 60 °C and −10 °C (40 °C and −30 °C), respectively^66^. The statistical algorithm identified records that are above the 3rd quartile plus *m* times the interquartile range (IQR) and those that are below the 1st quartile minus *m* times the IQR. For Tmax and Tmin, *m* was set to 3.5. It should be mentioned that the statistical algorithm was applied each month in order to take into account the seasonal cycle effect on the thresholds.
3. Internal consistency: inspection of daily records where Tmax is below Tmin. Furthermore, the values were flagged when Tmax and Tmin had the same magnitude (Tmax = Tmin).
4. Temporal coherence: inspection of daily values repeated over a long period and very extreme (day-to-day) jumps. It was defined that a value can be the same up to a maximum of 8 days. Additionally, a daily jump may not have a variation over 20 °C^67^.
5. Spatial coherence: comparison of the rank of each data value with the average rank of the data recorded at adjacent stations^68^. The original daily air temperature series were converted to percentiles. Each air temperature value was replaced by its corresponding percentile. For each time series, we selected the neighbouring stations which meet the requirements of being within 70 km and had an elevation difference of less than 500 m^69,70^. To perform the test, at least four neighbouring stations had to be available. If this was not the case, the daily value of the target station was not compared. The records of the target station with differences greater than a percentile of 0.85 concerning the average of the neighbouring stations were identified. The percentile difference approach allows for identifying only the most extreme spatial variations^71–73^.
6. Visual inspection: a visual inspection of the daily time series was carried out to identify periods with inhomogeneities that cannot be corrected (rounding errors, asymmetric rounding patterns, measurement precision, time irregularities, and obvious inhomogeneities)^51,74^. For this purpose, we used daily series and annual decimal frequency charts.

All QC-flagged values were set as a missing observation after the QC steps (Supplementary Figs. 1, 2). For the following procedures, only stations that retained the 5-year threshold after the QC were used. In addition, we manually verified the elevation information of weather stations using a digital elevation model (detailed in the *Spatial predictors for air temperature* sub-section) and modified it where necessary.

#### Gap-filling

Simple interpolation of incomplete data may produce artificial inhomogeneities in the gridded product due to the irregular spatiotemporal distribution of weather stations during the 1981–2020 period^75^. This can affect the variance and lead to erroneous conclusions on changes and variability^76^. To reduce such artifical inhomogeneities, data reconstruction of time series that do not cover the entire period and of gaps within time series was necessary.

A gap-filling procedure based on neighbouring stations^77^ was implemented to create a complete database. Before applying the algorithm, the available information was standardised using a daily climatology of the available data to avoid differences in the mean and the variance^78^. Subsequently, the model estimates were corrected to approximate the observed values as closely as possible. The correction was made by applying empirical quantile mapping^79,80^. The Tmax and Tmin series were reconstructed independently.

A neighbouring station was considered for gap-filling if it met two conditions: (i) at least five years of data in common, and (ii) a correlation greater than or equal to 0.6 with the target station. An iterative process of the gap-filling algorithm was performed to take advantage of those stations that did not have a common period at the beginning^81^. This was carried out in up to three iterations, where the availability of neighbouring stations was limited according to the following characteristics: horizontal-vertical distances of (i) 70 km–500 m, (ii) 100 km–500 m, and (iii) 150 km (no vertical limit), respectively. A maximum of 8 neighbouring stations was considered during this procedure. The rationale for this configuration was based on a previous correlation-distance-elevation analysis (Supplementary Fig. 3).

Due to the low density of weather stations in some regions, virtual stations (time series at the closest grid point) from the ERA-5 Land reanalysis^82^ were additionally included to fill temporal gaps. These time series were not directly used, but an anomaly-based bias correction (detrended empirical quantile mapping^83^) was applied to series with at least ten years of data. Only those virtual stations with a correlation greater than or equal to 0.6 with the target station (within Peru) were preserved and used for gap-filling.

#### Homogenisation

Many non-climatic influences can affect measurements (changes in station location, instrumentation, and observing practices, among others). To eliminate these inhomogeneities and to obtain more reliable observations, time series must be homogenised^84,85^. A variety of statistical methods has been developed, each with different results^84,86^. In sparse networks, homogenisation performance is drastically reduced, and there is a risk of erroneous corrections due to the low signal-to-noise ratio^87^. Consequently, the chosen method must be applied carefully.

We tested the temporal homogeneity using the Standard Normal Homogeneity Test^88,89^ in both its relative form, known as the Pairwise Homogeneity Algorithm (PHA)^90,91^, and its absolute implementation. The process was fully automatic and straightforward. Therefore, the approach was consistent, unlike semi-automatic approaches that require several subjective decisions that can influence the whole process^74^. In addition, PHA has been applied at global scale datasets^92,93^, and is one of the approaches with the best performance^84,86^.

The algorithm searched a maximum (minimum) of eight (four) neighbouring reference stations with a correlation greater than or equal to 0.6 with the target station within a horizontal (vertical) distance of 1000 km (1000 m) in order to perform a relative test. In absence of these conditions, the absolute test was applied. Absolute tests have a lower detection efficiency than relative tests^84^. Therefore, the condition was designed as a backup test when a relative test was almost impossible to apply^94^. In both cases, a *p*-value < 0.05 (with a 95% confidence interval) was used to define significant breakpoints which were then used to adjust past values compared to the present.

As the algorithm was applied on a monthly scale, a linear time interpolation of the monthly correction factors to a daily scale was performed^95^. The homogeneity tests were applied after the gap-filling to (i) detect inhomogeneities introduced by the gap-filling process, and, (ii) because the process was more reliable if the time series had no gaps^50,71^. Finally, as for the gap-filling procedure, homogenisation was performed in up to three repetitive cycles according to the boundary conditions previously defined.

### Spatial predictors for air temperature

In the gridding process, Tmax and Tmin were adjusted to a series of auxiliary spatial predictors such as land surface temperature (LST), elevation (DEM), latitude (Y), longitude (X), and the topographic dissection index (TDI).

The LST observations were selected from MODIS^96^. This satellite product provides average 8-day values starting in the year 2000 and at a 1 km spatial resolution. The Terra version (MOD11A2 V6)^97^ was used for day (LST_day) and night (LST_night) observations. Because of missing data before 2000, the average monthly values for 2000–2020 for both day and night times were used as spatial predictors for Tmax and Tmin, respectively. Only LST values were used without cloud contamination, emissivity error >0.02, or LST errors >2 °C. If any grid cell in the final average were empty, they were reconstructed through nearest neighbour interpolation. The LST was downloaded from https://developers.google.com/earth-engine/datasets/catalog/MODIS_006_MOD11A2 (accessed 31 October 2022).

The DEM data were obtained from the Global Multi-resolution Terrain Elevation Data (GMTED) 2010^98^ at a spatial resolution of 1 km. This dataset was selected because it has also been used in other temperature-gridded products at a national level^38^. X, Y, and TDI were derived at the same spatial resolution as the DEM. The digital elevation model was downloaded from https://developers.google.com/earth-engine/datasets/catalog/USGS_GMTED2010 (accessed 31 October 2022).

The TDI was calculated through a multi-scale DEM calculation:

1 $$TD{I}_{({s}_{0})}=\mathop{\sum }\limits_{i=1}^{n}\frac{Z({s}_{0})-{Z}_{min}(i)}{{Z}_{max}(i)-{Z}_{min}(i)}$$

Where $TD{I}_{({s}_{0})}$ is the final multi-scale TDI value for the grid cell location *s*_0_, $Z({s}_{0})$ is the elevation at the grid cell location *s*_0_, ${Z}_{min}(i)$ is the minimum elevation at the grid cell location in the spatial window *i*, ${Z}_{max}(i)$ is the maximum elevation at the grid cell location in the spatial window *i*, and *n* is the number of spatial windows^99^. The TDI value for a specific window size represented the height of a grid cell relative to the surrounding terrain. The multi-scale TDI was calculated for five spatial window sizes (at 3, 6, 9, 12, and 15 km). Valley bottoms and low areas relative to surrounding grids have values close to zero, while ridges and areas above surrounding areas have high values approaching 5. The selection of this topographic variable was based on the high correlation with daily Tmin anomalies which are influenced by cold air drainage^13,99^.

The spatial predictors were downloaded from the Earth Engine Data Catalog^100^ repository via rgee^101^. For efficient processing, the data were adapted to the extent of −81.405°, −67.185°, −18.595°, and 1.225° (min longitude, max longitude, min latitude, and max latitude); and re-gridded at 0.01° spatial resolution.

### Air temperature interpolation

For the interpolation of Tmax and Tmin, a climatologically aided interpolation (CAI) approach^59–62^ was used. With CAI, deviations from the average (anomalies) on a given day were interpolated and combined with an average field (climatology) to produce the final daily product. The CAI approach has been employed in several studies^13,18,62,73^ and has proven to be effective to improve the accuracy of air temperature estimation in regions of complex terrain with limited observations^102–105^. This approach drastically reduced computational costs compared to independent runs for each time step, and the co-variables did not necessarily need to be in the same temporal range as the observational data. The procedure was applied independently for Tmax and Tmin and comprised three steps:

1. Interpolation at monthly (normal) average scale for the 1981–2010 period.
2. Interpolation at the daily anomaly scale (based on the monthly normal) for 1981–2020 period.
3. Combination of 1 and 2 to obtain the daily temperature value.

#### Monthly normal interpolation

For the interpolation of the monthly normal, the Regression-Kriging (RK) method^13,29,106^ was used, which represents a spatial process expressed as the sum of a deterministic and a stochastic part:

2 $$\overline{T}({s}_{0},{m}_{0})={\overline{T}}_{u}({s}_{0},{m}_{0})+{\overline{T}}_{e}({s}_{0},{m}_{0})$$

Where $\overline{T}({s}_{0},{m}_{0})$ is the final interpolated normal temperature at the grid cell location *s*_0_ and for the month *m*_0_, ${\overline{T}}_{u}({s}_{0},{m}_{0})$ is the deterministic spatial trend in normal temperature modelled by the weather station locations and auxiliary predictors, and ${\overline{T}}_{e}({s}_{o},{m}_{o})$ is the spatially autocorrelated stochastic residual with zero mean^107^. We use a linear model to fit ${\overline{T}}_{u}({s}_{0},{m}_{0})$, and ordinary kriging (OK) to interpolate the residual part ${\overline{T}}_{e}({s}_{o},{m}_{o})$:

3 $$\overline{T}({s}_{0},{m}_{0})={\beta }_{0}+{\beta }_{1}lst({m}_{0})+{\beta }_{2}z+{\beta }_{3}x+{\beta }_{4}\,y+\mathop{\sum }\limits_{i=1}^{n}{w}_{i}({s}_{0},{m}_{0}){\overline{T}}_{e}({s}_{i},{m}_{0})$$

*β*_0_ is the intercept; *β*_1_, *β*_2_ *β*_3_ and *β*_4_ are the model coefficient estimates for monthly average LST, elevation, latitude, and longitude, respectively; $lst({m}_{0})$, *z*, *x* and *y* are the average LST at *m*_0_, elevation, longitude, and latitude at grid level at the location *s*_0_; ${w}_{i}({s}_{0},{m}_{0})$ are the weights defined by the residual spatial covariance; and ${\overline{T}}_{e}({s}_{i},{m}_{0})$ are the residuals of the regression for *n* stations.

Due to the large variability and extent of the study area, it was not appropriate to use a global model for the spatial prediction of normal temperature. A version of RK with a moving spatial window based on Geographically Weighted Regression-Kriging (GWRK)^108^ was used to account for the spatial heterogeneity in the interpolation process. The GWR^109,110^ calculated local trends for a subset of the study area with a weighting of weather stations using a distance-based function. To improve prediction accuracy, it added the OK from the residuals to the regression estimate. The weighting of the observations in GWR was calculated using the bi-square kernel nearest neighbourhood function:

4 $${w}_{i}({s}_{0})={\left[1-{\left(\frac{h{({s}_{0})}_{i}}{r}\right)}^{2}\right]}^{2}$$

Where *w*_*i*_(*s*_0_) is the distance-based weighting function of the station *i* at the interpolation location *s*_0_, *h*(*s*_0_) is the distance between the station *i* and the interpolation location *s*_0_, *r* is the bandwidth for the size of the spatially adaptive kernel function. The bandwidth optimisation was necessary because a significant deviation in estimating the regression parameters would be generated if the bandwidth were too large or too small^109^. The Corrected Akaike Information Criterion automatically determined the optimal bandwidth^110^.

The regression coefficients of the GWR model were estimated at a spatial resolution of 0.1°, assuming that the relationship between the normal temperature and the auxiliary predictors is independent of the spatial resolution scale^111,112^. Then it was locally interpolated with a bilinear approach at a resolution of 0.01° to be applied to the auxiliary predictors. The OK of the residuals was set to 0.05° and then disaggregated to 0.01° to reduce the measurement precision inconsistencies^51,113,114^ of the observed time series (Supplementary Fig. 4). Both sub-products at the final resolution were aggregated according to Eq. 3 to obtain the grids of the monthly normals of Tmax and Tmin.

We used the GWmodel^110^ and gstat^115,116^ packages for the implementation of GWRK. For the estimation of the theoretical variogram (in OK), an automatic adjustment by iteratively repeated minimum squares was used, and the nugget value was forced to zero according to the automap package^117^.

#### Daily interpolation

A method similar to the monthly normal temperature was used in the daily temperature interpolation. In this sense, the daily anomalies of Tmax and Tmin were expressed as the sum of two components (deterministic and stochastic). Because of the large number of days (14244) per variable and the intention to produce PISCOt operationally, it was chosen to use RK due to computational limitations. The model here was similar to Eq. 3 but added the spatial predictor TDI.

Therefore, the daily temperature product was obtained according to:

5 $$T({s}_{0},{d}_{0})=\overline{T}({s}_{0},{m}_{0})+\delta T({s}_{0},{d}_{0})$$

Where $T({s}_{0},{d}_{0})$ is the temperature at the interpolation point *s*_0_ for the day *d*_0_ within the month *m*_0_, $\overline{T}({s}_{0},{m}_{0})$ is the normal temperature in the month *m*_0_ according to Eq. 3, and $\delta T({s}_{0},{d}_{0})$ is the daily temperature anomaly at the interpolation point *s*_0_ for the day *d*_0_.

Unlike traditional CAI applications, we employed spatial predictors in $\overline{T}({s}_{0},{m}_{0})$ and $\delta T({s}_{0},{d}_{0})$^13,39^. Some research have found that topographic factors in a mountainous region are directly related to the spatial patterns of $\delta T({s}_{0},{d}_{0})$, particularly during stable atmospheric conditions that favour cold air inversion^13,99^.

## Data Records

The generated dataset consists of gridded, geo-localised files and a chart presenting information on the weather stations used. For quick access, the data are divided into different repositories (Table 1) and are stored in a figshare collection^118^ (10.6084/m9.figshare.c.5959863).

**Table 1:** Accession and data files for each repository of the database.

Order	Repository Name	Data	Format	Number of files	Files	Access
1	Maximum temperature (PISCOt v1.2)	Tmax	.nc	41	tmax_mean_1981-2010.nc, tmax_daily_1981.nc, ⋮ tmax_daily_2020.nc	10.6084/m9.figshare.20522806
2	Minimum temperature (PISCOt v1.2)	Tmin	.nc	41	tmin_mean_1981-2010.nc, tmin_daily_1981.nc, ⋮ tmin_daily_2020.nc	10.6084/m9.figshare.20533715
3	Spatial Covariables for PISCOt v1.2	DEM, Y, X, TDI, LST_day, LST_night	.nc	6	DEM.nc, Y.nc, X.nc, TDI.nc, LST_day.nc, LST_night.nc	10.6084/m9.figshare.15167517
4	Weather stations used in PISCOt v1.2	list of weather stations	.csv	1	xyz_qc_gf_hmg. csv	10.6084/m9.figshare.14329208
5	Maximum and Minimum temperature at a coarser resolution (PISCOt v1.2)	Tmax, Tmin	.nc	8	tmax_mean_1981-2010_005.nc, tmin_mean_1981-2010_005.nc, tmax_daily_1981_2020_005.nc, tmin_daily_1981_2020_005.nc, tmax_mean_1981-2010_010.nc, tmin_mean_1981-2010_010.nc, tmax_daily_1981_2020_010.nc, tmin_daily_1981_2020_010.nc	10.6084/m9.figshare.22712365

The files of normal (average) and daily Tmax and Tmin values are stored in Repository 1 and 2, respectively. These data represent the primary output of the research (a gridded 0.01° spatial resolution product, PISCOt v1.2) and are available in the Network Common Data Form (NetCDF) format. Normal values are stored in a single file whereas daily values are stored in different archives divided by year from 1981 to 2020.

The files of the spatial covariables are stored in Repository 3. These represent the predictors (X, Y, DEM, LST_day, and LST_night) used to build the spatial models of Tmax and Tmin and are available in NetCDF format.

The list of all weather stations used as input for PISCOt v1.2 is stored in Repository 4. The file contains the following information (headers): code (*ID*), name (*NAM*), longitude (*LON*), latitude (*LAT*), elevation (*ALT*), and source (*SRC*) of each weather station. In addition, it also provides information if a weather station has been selected as a virtual station (bias-correction of ERA5-Land) in the gap-filling procedure (*filter_qc*); and, if a weather station has been used for cross-validation in the gap-filling procedure and daily spatial model (*filter_qc70*). The file is available in Comma Separated Values (CSV) format.

The gridded product of PISCOt v1.2 was also produced at a coarser spatial resolution (at 0.05° and 0.10°) using the same methodology and input data. This dataset is available in Repository 5. The purpose to provide these different versions is to facilitate quick access to the data of Tmax and Tmin as the original version (0.01°) includes large file sizes. The normal and daily values of Tmax and Tmin at 0.05° and 0.10° spatial resolution are stored in single NetCDF files.

The data in each NetCDF file consists of three dimensions (*time*, *latitude*, and *longitude*). For monthly normal files, the *time* dimension corresponds to the month of the year beginning with January. Each repository in Table 1 provides in addition a README file with a brief explanation of the dataset. Finally, Repositories 1 and 2 will also be available as a secondary repository in the Google Earth Engine Data Catalog.

## Technical Validation

The development process of PISCOt has been evaluated in three steps: (i) gap-filling validation; (ii) spatial model validation; and (iii) usefulness of the PISCOt product. In the spatial model validation, we focused on the assessment at monthly normal and daily scales. In the usefulness of the PISCOt product, we provided two applications, one associated with spatio-temporal variability of air temperature, and the other related to the coastal fog effect on air temperature.

The statistics used to evaluate the skill of each step were simple error (mean bias), mean absolute error (MAE), and the refined index of agreement (*d*_*r*_)^119^. The *d*_*r*_ metric ranges from −1.0 to 1.0, with a value of >0.5 indicating a higher predictive capacity than the observed average. Because the primary mode of variability in air temperature is usually the seasonal cycle, the metrics were calculated independently for each month and then averaged. This baseline adjustment in *d*_*r*_ prevented from overestimating the skill of each reconstruction (i.e. gap-filling, etc.) by correcting for the seasonal cycle^120^. Furthermore, the non-parametric Mann-Kendall test associated with Sen’s slope estimator was used for trend analysis in the evaluation.

### Gap-filling validation

A gap-filling procedure was applied to extend shorter times series of weather stations (back to 1981) before constructing PISCOt. Two analyses were conducted to evaluate the efficiency of the gap-filling procedure. (i) Validation: comparing infilled and observed data for available dates with observed values, i.e., comparing available data that has been used to build the model. (ii) Cross-validation: comparing infilled and observed data for dates that were artificially set as missing data, i.e., comparing data that has not been used to build the model. In cross-validation, it is assumed a worst-case missing data scenario, we set only ten years of data in stations with more observed data (in time series with ≥75% of non-missing data in the period 1981–2020).

**Fig. 3:**
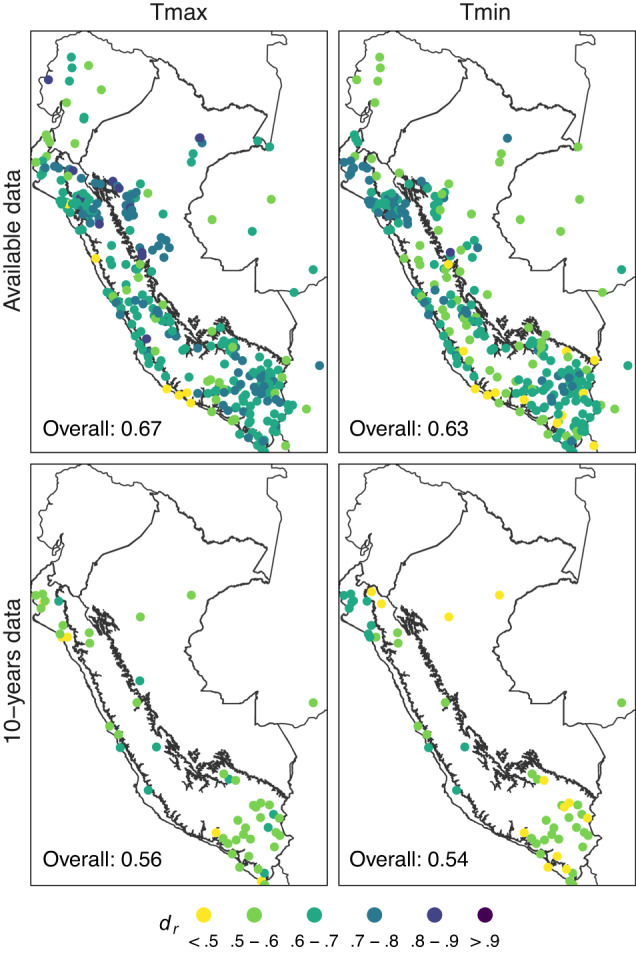
Spatial distribution of the refined index of agreement (*d*_*r*_) for gap filling (1981–2020) of daily maximum (Tmax) and minimum (Tmin) air temperature in two experiments: validation, using all available data; and, cross-validation, when only a complete period of 10-years (with ≥75% data) is available. Black lines represent the three main climate regions: Pacific Coast (Western Peru), Andes (Central Peru), and Amazon (Eastern Peru).

Table 2 summarises the statistical metrics, and Fig. 3 shows the distribution of *d*_*r*_ for both experiments. The experiments showed that the efficiency was slightly better for Tmax than Tmin. Both experiments had a bias <0.2 °C and MAE <1.5 °C. The most significant difference was in *d*_*r*_; although moderate-to-high efficiency values were obtained in both experiments (*d*_*r*_ > 0.5), the best results were obtained in experiment (i). This can be explained due to the small amount of information available in the experiment (ii), as it was a worst-case scenario. By visualising the spatial distribution of *d*_*r*_, it was noted that there were higher (lower) values in more (less) dense regions of weather stations for both experiments. The areas where *d*_*r*_ reached values from 0.8 to 0.9 were found in experiment (i). On the other hand, in experiment (ii), it reached values from 0.6 to 0.7.

**Table 2:** Gap filling error statistics for daily maximum (Tmax) and minimum (Tmin) temperature for bias, mean absolute error (MAE), and refined index of agreement (*d*_*r*_) for 1981–2020 in two experiments: using all available data and when only a complete period of 10-years (with ≥75% data) is available.

Experiment	Tmax				Tmin			
	Number of stations	bias (°C)	MAE (°C)	*d*_*r*_	Number of stations	bias (°C)	MAE (°C)	*d*_*r*_
Validation (Available data)	346	0.11	0.98	0.67	342	0.1	0.98	0.63
Cross-validation (10-years data)	51	0.03	1.27	0.56	52	−0.02	1.44	0.54

In general, the validation errors showed that the here-in used infill models worked reasonably well, considering the complicated topographic variability of the study area and the limited observational data. It must be pointed out that the errors of experiment (i) represented the residuals between the filled and observed values, as these were used to construct the infilled models that were finally used in PISCOt.

### Spatial model validation

#### Monthly normal air temperature

K-fold cross-validation was performed to characterise the efficiency of the spatial model for the monthly normal temperature. In this study, K = 10 was defined. Therefore, 10 clusters were set up for each model and data series. We applied the statistical metrics (bias and MAE were only used as they are less affected by sample size) at the scale of two seasonal periods: “warm” (October to March) and “cold” (April to September).

**Fig. 4:**
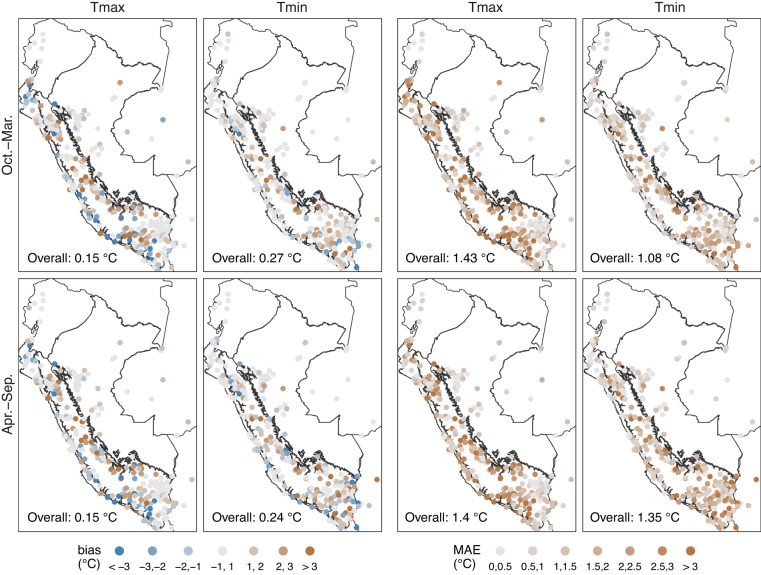
10-fold cross-validation bias and mean absolute error (MAE) for interpolated monthly maximum (Tmax) and minimum (Tmin) normal temperature in the period 1981–2010 (*n* = 299 stations). Black lines represent the three main climate regions in Peru (Fig. 3).

Figure 4 showed a smaller positive bias in Tmax than in Tmin, with an average (warm and cold) value of 0.15 °C and 0.25 °C, respectively. However, this may be biased due to negative errors in the average. Considering the biases at the station scale, more points fall within the range of −1 °C to 1 °C in Tmin, implying that the estimation was better for Tmin. This pattern confirmed the findings for MAE, where Tmin (Tmax) averages 1.22 °C (1.42 °C) for both seasons. Spatially, the monthly normal interpolation performed worst in the mountainous regions between the boundaries of the climatic regions (Pacific Coast - Andes and Andes - Amazon), mainly in Tmax. Similarly, the largest errors in Tmax can be found in the southern Pacific Coast. At the seasonal level, there was no considerable difference in Tmax. However, for Tmin, estimates were slightly better in the warm period than in the cold period.

**Fig. 5:**
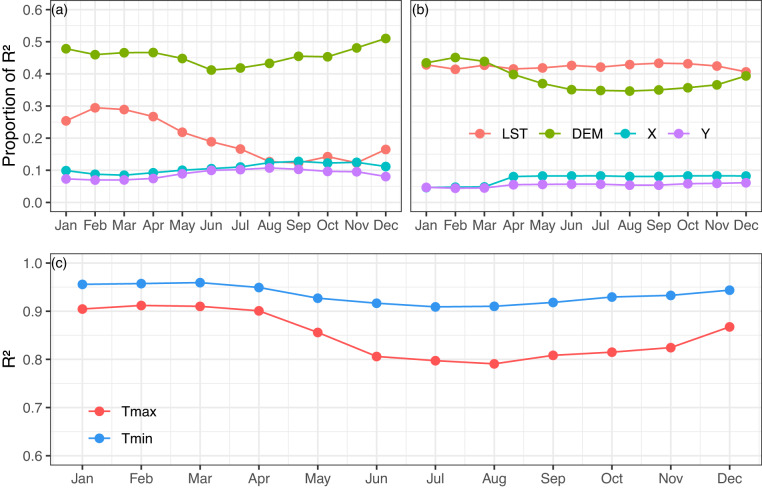
Relative and absolute influence of spatial predictors (land surface temperature (LST), elevation (DEM), latitude (Y), and longitude (X)) over Peru using a monthly-normal moving window with multiple linear regression relating the Geographically Weighted Regression Kriging (GWRK). Proportion of variance explained (R^2^) of each predictor for (**a**) maximum air temperature (Tmax) and (**b**) minimum air temperature (Tmin); and, (**c**) overall R^2^. The statistical values are averaged over 253 stations.

These results showed that the monthly normal interpolation for Tmin tends to be more efficient than for Tmax. In order to understand the impact of the spatial predictors (LST and DEM) on the air temperature estimation, the Lindemann, Merenda, and Gold method was applied^13,121,122^. This method quantifies the relative influence of a spatial covariate by partitioning the total variance explained by the R^2^ of the model (Fig. 5).

In Tmax (Fig. 5a), the DEM had the highest relative importance. The DEM contributed slightly more in summer than in winter months. About 50% (40%) of the observed variance can be explained by DEM in summer (winter). LST, on the other hand, adopts a major role from summer to autumn rather than during the period from winter to spring. One probable reason why DEM was such a good predictor for Tmax is that Tmax generally has a decreasing simple linear relationship with DEM, and DEM already has a solid predictive capacity without the addition of LST^13,28,47,123^. In addition, LST_day is highly influenced by incoming solar radiation and biophysical properties (e.g. land cover, albedo, moisture, roughness) and, thus, has a high degree of microscale variability^124^. As a result, LST_day is more spatially variable than Tmax, especially during higher solar radiation dates^47^. The relationship between Tmax and LST is often more complex than that between Tmin and LST^13,47^. From a seasonal perspective, we found that LST_day is more efficient in explaining the variance of Tmax from summer to autumn rather than winter to spring. We hypothesize that this behaviour can be related to solar radiation seasonality which is coupled with the cloud cover amount due to the rainfall season^42,75^. From winter to spring (summer to autumn) there is more (less) incoming solar radiation due to the presence of less (more) cloud cover. Consequently, the spatial relation between LST_day and Tmax is weaker in the winter season compared to the summer period.

For Tmin, LST was a slightly more critical predictor than DEM in most months except for February (Fig. 5b). However, no covariate reached a relative importance of 50%. It is somewhat notable that LST reached its highest values from June to November and, inversely, in DEM. Due to the strong gradients and complex topography, micro-climatic influences on Tmin play an essential role. Cold air inversions are a common phenomenon, especially during periods of atmospheric stability and significant radiative cooling which is typical for mountainous regions^28,99^. Therefore, Tmin does not have a simple linear relationship with DEM, which can limit its capacity as an individual predictor for the spatial patterns of Tmin^125^. The addition of LST, however, contributed to the spatial estimation of Tmin. Unlike LST_day, without direct solar radiation LST_night spatial variability is more influenced by local and mesoscale atmospheric processes important for air temperature^124^. Therefore, LST_night and Tmin maintain similar spatial variability throughout the annual seasonal cycle as contrary to LST_day and Tmax^13,47,126^. This is also shown by the fact that higher values of R^2^ were reached with Tmin (Fig. 5c) than with Tmax.

In summary, it was shown that the spatial model used had a greater predictive capacity and a lower average error in the estimation of Tmin than Tmax, mainly during the summer months. LST had a higher value-added in Tmin than in Tmax in the study region. Furthermore, DEM was more important for Tmax prediction.

#### Daily air temperature

The evaluation of the efficiency of daily air temperature data was similar to the one presented for the monthly normals, but only focused on the stations with long time series (with ≥75% of non-missing data) to reduce the influence of synthetic data. In addition, trends (Sen’s slope) were computed over the available period for each station and were compared with trends calculated based on the 10-fold cross-validation. This analysis allows to estimate how reliable temperature trends can be predicted at un-sampled locations by interpolation, giving insight into the accuracy of temperature trends from the gridded dataset^16,127^.

**Fig. 6:**
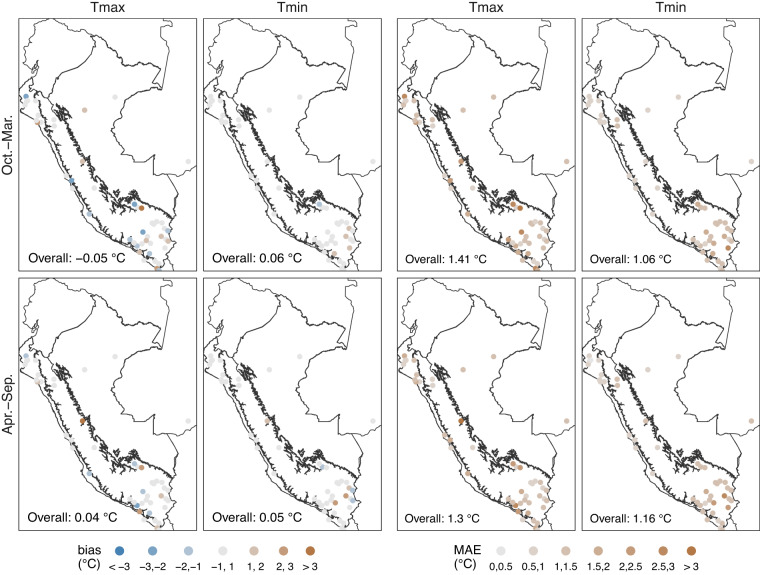
10-fold cross-validation bias and mean absolute error (MAE) for interpolated daily maximum (Tmax) and minimum (Tmin) temperature in the period 1981–2010 (*n* = 48 stations). Black lines represent the three main climate regions in Peru (Fig. 3).

**Fig. 7:**
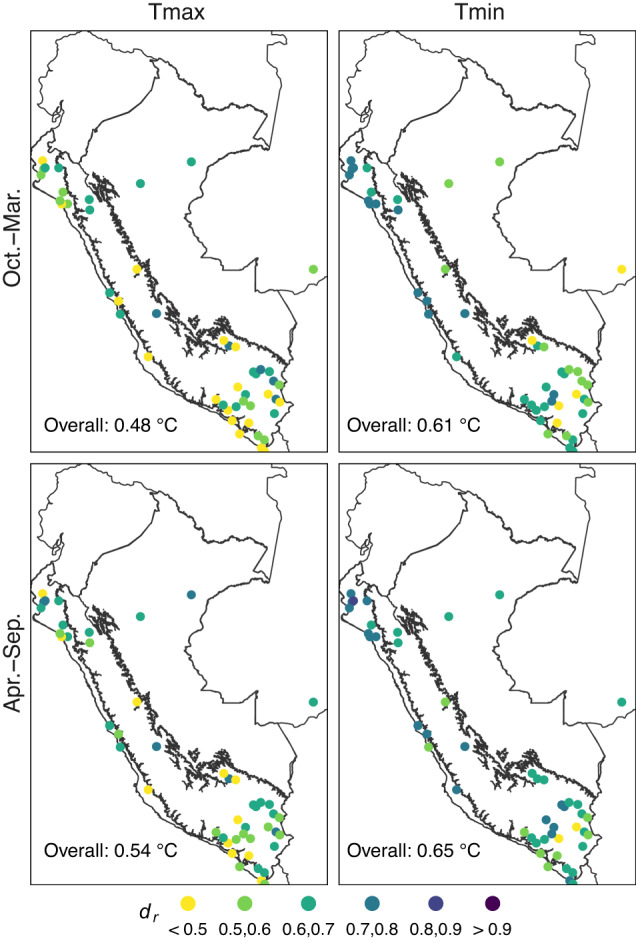
10-fold cross-validation refined index of agreement (*d*_*r*_) for interpolated daily maximum (Tmax) and minimum (Tmin) temperature in the period 1981–2010 (*n* = 48 stations). Black lines represent the three main climate regions in Peru (Fig. 3).

Figure 6 shows the results for bias and MAE, while Fig. 7 shows the results for *d*_*r*_. On average, a lower bias was observed compared to the normal scale. This was probably due to the greater amount of averaged data. Despite this, it can be observed that there was a similar pattern to the normal scale. For the bias (MAE), values of −0.01 °C and 0.05 °C (1.36 °C and 1.11 °C) were found on average for Tmax and Tmin, respectively. Furthermore, estimates were slightly better for Tmax (Tmin) in the cold (warm) period. For *d*_*r*_, it reached moderate-to-high efficiency values (*d*_*r*_ > 0.5) at most of the weather stations. Efficiency values were lowest for the warm period of Tmax (*d*_*r*_ = 0.48). The area with the lowest *d*_*r*_ values was in the south, mainly along the Pacific Coast and the border regions of the Andes and the Amazon.

**Fig. 8:**
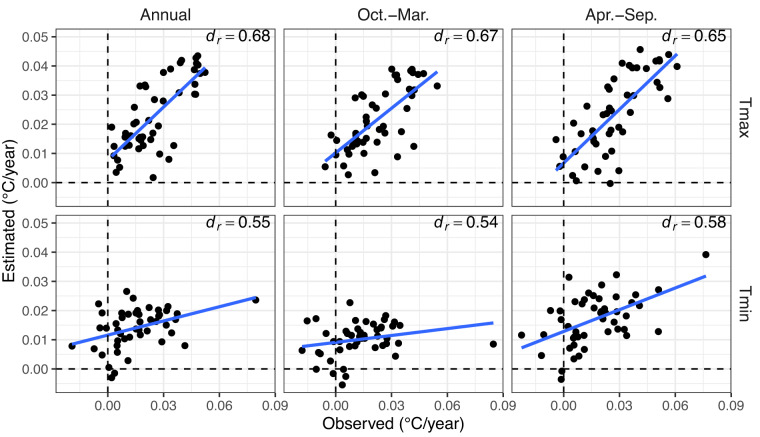
Scatterplot of estimated (10-fold cross-validation) and observed air maximum (Tmax) and minimum (Tmin) temperature trends (Sen’s slope) in the period 1981–2020. Mean values at annual; and October-March and April-September mean seasons. Blue lines (linear regression) show the agreement between observed and estimated trends. The refined index of agreement (*d*_*r*_) is also shown for each plot (*n* = 48 stations).

Figure 8 exhibits the cross-validated predictions of the 1981–2020 trends in the annual mean and the warm and cold seasons of air temperature with the trends observed in the homogenized weather stations. This shows that most signs of observed trends are well detected by the estimated time series, particularly for Tmax rather than Tmin. The disparity is also evidenced by the *d*_*r*_ metric, where estimated trends for Tmax are above 0.6 while for Tmin they are around 0.5–0.6. There is not much difference between the annual and seasonal means. The results indicate that there is moderate efficiency in reproducing the observed spatial variations of the temporal trends in Tmax, but for Tmin, there is a poorer capability. This is probably due to the limited station density in Peru and artificial temporal variability mixed with real local climate features, despite the homogeneity check and QC procedures^16^. For the case of Tmin, the low temporal variability estimation can also be attributed to the lower temporal correlation power at shorter distances compared to Tmax (Supplementary Fig. 3), leading to a less efficient temporal reconstruction (as shown in the *Gap-filling validation* sub-section), and hence a lower temporal variability estimation. Furthermore, as Tmin is more influenced by local conditions, there would be a possible role of land cover change that has not been taken into account as a predictor^128,129^. Finally, it is worth mentioning that LST did not cover the entire period, which could also explain the bad performance of temporal trends.

In general, the results demonstrated a reasonably good capacity of the spatial model to estimate daily Tmax and Tmin. Similarly to the results from the normal monthly scale, Tmin outperformed Tmax in both the warm and cold periods. However, Tmax is slightly more efficient in estimating the observed spatial variations of the temporal trend.

### Usefulness of the PISCOt v1.2 product

#### Spatio-temporal variability of air temperature

To present an application of PISCOt v1.2, a description of the spatio-temporal variability of air temperature indices characterising the trend (Mann-Kendall test and Sen’s slope) was conducted. This was applied in the southern Andes of Peru, a region characterised by agricultural and livestock subsistence and production^42^, and therefore highly dependent on climatic conditions. The indices selected were annual mean Tmax (MTmax), annual mean Tmin (MTmin), and the annual number of frost days (FD, number of days with Tmin <0 °C).

Additionally, to provide a full comparison with existing temperature products, both national datasets (PISCOt v1.1 and VS2018, described above) and global products (TerraClimate^130^, CHIRTS^9^, and ERA5-Land^82^) were included. TerraClimate provides Tmax and Tmin at monthly temporal resolution and a ≈4 km spatial resolution for 1958–2020. CHIRTS produces daily values of Tmax and Tmin at 5 km (0.05°) and is available from 1983 to 2016. ERA5-Land is a reanalysis product that contains a great diversity of surface variables at a spatial resolution of 9 km (≈0.1°) since 1981. For ERA5-Land, daily Tmax and Tmin were obtained from the maximum and minimum hourly values.

**Fig. 9:**
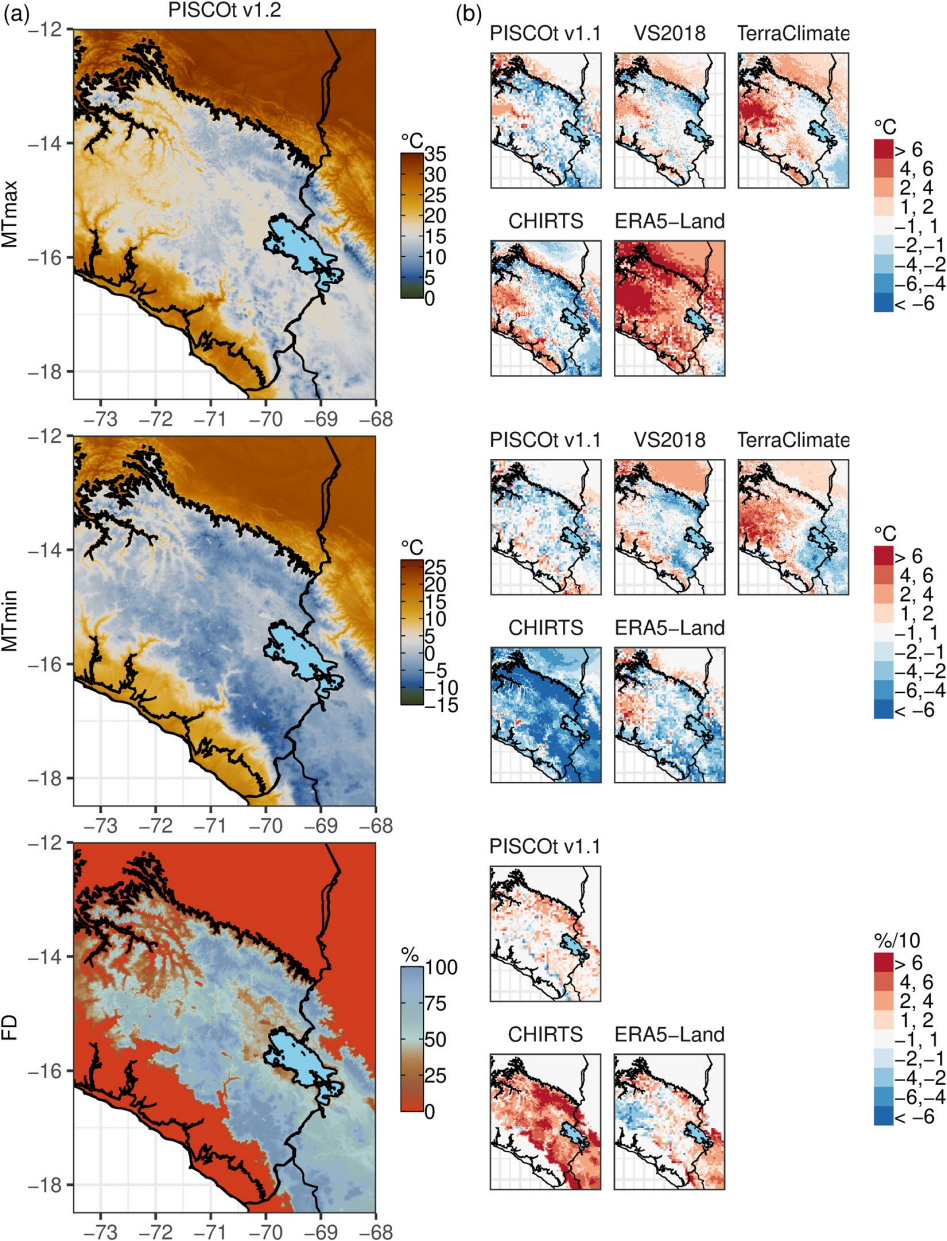
Spatial distribution and differences of the mean annual (1981–2010) temperature indices (mean Tmax (MTmax), mean Tmin (MTmin), and frost days (FD)) in the southern Andes of Peru. (**a**) Spatial distribution for PISCOt v1.2. (**b**) Difference of PISCOt v1.2 with each gridded product (PISCOt v1.1, VS2018, TerraClimate, CHIRTS, and ERA5-Land) and temperature indices. For CHIRTS, the mean average corresponds to 1983–2010. Black lines represent the three main climate regions in Peru (Fig. 3); Lake Titicaca is shown as a lightblue filled area.

First, the spatial differences for the annual average air temperature indices were examined for the period 1981–2010. Figure 9a shows the annual climatologies of MTmax, MTmin, and FD in PISCOt v1.2, while Fig. 9b indicates the difference of PISCOt v1.2 with each gridded product. For MTmax, differences were small (below 1 °C), mainly in PISCOt v1.1 and VS2018. ERA5-Land presented the lowest MTmax values compared to PISCOt v1.2, reaching differences of up to more than 6 °C in large parts of the Andean and Amazonian regions. The largest areas of differences between the multiple gridded products occured at the boundaries of the climatic regions, i.e., at the Andes-Amazon and Pacific-Andean transitions and where no data were available. For MTmin, the spatial pattern of the differences was similar to MTmax for PISCOt v1.1 and VS2018. The largest differences were found in TerraClimate and CHIRTS, where the latter had the highest MTmin values, reaching differences of up to more than −6 °C in the Andean highlands. For FD, PISCOt v1.1 and ERA5-Land showed the best agreement with PISCO v1.2 (differences within 10%). Only for CHIRTS, differences of up to 60% were discovered. This was not surprising as CHIRTS represents the most diverging product regarding Tmin.

**Fig. 10:**
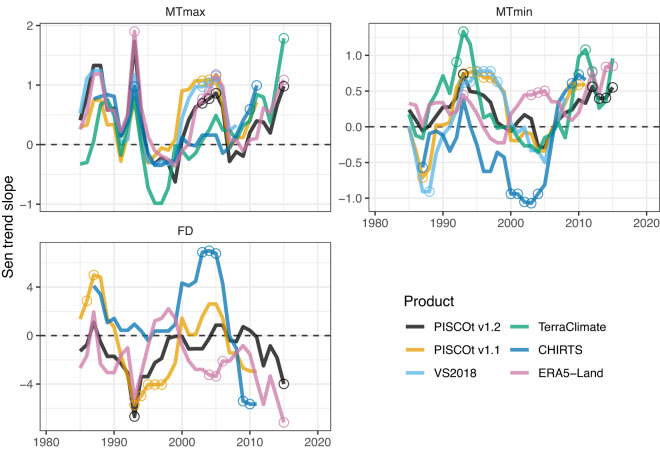
Running annual Sen’s slope (for 10-years window from 1981 to 2020) of temperature indices (mean Tmax (MTmax), mean Tmin (MTmin), and frost days (FD)) for PISCOt v1.2 and gridded products (PISCOt v1.1, VS2018, TerraClimate, CHIRTS, and ERA5-Land) in the southern Andes of Peru (following delimitation of red box in Fig. 2 considering all land >2000 masl). Significant trend estimates (Mann–Kendall trend test with *p* < 0.05) are shown with an open circle. The x-axis shows the centroid year of running trends.

The spatio-temporal variability of air temperature indices was assessed through trend analysis at different temporal and spatial windows. Figure 10 shows the decadal rate of change for 10-year time windows from 1981 to 2020 for areas above 2000 masl in the Southern Andes of Peru. For MTmax, there was a good agreement between the trends of the different products. Periods with significant positive trend were coinciding well in all products in the 1990–1995, 2000–2005, and 2010–2015 years. Periods with slightly negative or zero trends coinciding well in all products in the 1995–2000 and 2005–2010 years. This was evident in PISCOt v1.2 compared to ERA5-Land, VS2018, and PISCOt v1.1. For MTmin, there was more variability in the trends, with no clear overall direction as in MTmax, except for the latest years (since 2010). From 1980 to 2000, PISCOt v1.2 showed similar variability (a slightly positive trend) to ERA5-Land, then moves closer (a slightly negative trend) to PISCOt v1.1 and VS2018 in the 2000–2007 period, and finally, since 2010, being in agreement with PISCOt v1.1 and VS2018 and ERA5-Land into a positive trend. It is worth noting that PISCOt v1.1 and VS2018 showed good agreement in Tmin throughout the analysis period, diverging to a greater extent from PISCOt v1.2 before 1990. Significant positive trends in common in MTmin were only found during 1990–1995 and 2010–2015. A similar pattern as for MTmin was also found for FD. ERA5-Land (PISCOt v1.1) tended to behave analogously to PISCOt v1.2 for much of the analysis period, only disagreement (agreement) from 1995 to 2007. There were only significant overlapping trends in FD during 1990–1995 (negative) and 2010–2015 (positive).

**Fig. 11:**
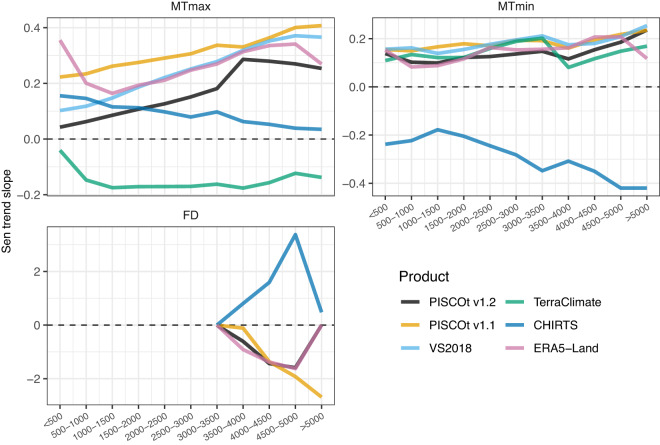
Annual Sen’s slope (1983–2013) of temperature indices (mean Tmax (MTmax), mean Tmin (MTmin), and frost days (FD)) per different elevations intervals (km asl.) for PISCOt v1.2 and gridded products (PISCOt v1.1, VS2018, TerraClimate, CHIRTS, and ERA5-Land) over the southern Andes of Peru.

Regarding spatial variability, Fig. 11 shows the trend by different elevation intervals for the period 1983–2013 (common reporting period). In MTmax, the magnitude of trends increased for higher elevation intervals mainly in PISCOt v1.2, PISCOt v1.1, VS2018, and ERA5-Land. In contrast, in CHIRTS and TerraClimate no direct relationship between the elevation and trend magnitude was evident. There was a more substantial spatial disparity in the direction of the trends at lower than high elevations in the different products (Supplementary Fig. 5). For MTmin, the various products (except for CHIRTS) showed a better agreement of the relationship between the trend magnitude and elevation. However, this was less pronounced than for MTmax. Significant positive or negative trends in FD were only found between 3000 and 3500 masl, with a similar (inverse) agreement of PISCOt v1.2 with PISCOt v1.1 and ERA5-Land (CHIRTS). PISCOt v1.2 and ERA5-Land reached zero trends above 5000 masl, because for this elevation level for every year 100% FD was reached. Consequently, no temporal change can be found.

The results showed that PISCOt v1.2 performed well over the southern Andes of Peru. PISCOt v1.2 presented spatiotemporal trends and overall distribution similar to the other products. Some differences in the results can be pointed out. Firstly, there was a high degree of correspondance in the magnitude of the air temperature between PISCOt v1.2 and PISCOt v1.1 and VS2018. This was expected, since the three datasets used information from the same station’s network, albeit with a different number of stations and distinct pre-processing applied. Larger differences were obtained in ERA5-Land (MTmax) and CHIRTS (in MTmin and FD). ERA5-Land is a reanalysis-based dataset, thus, it is expected to represent the physics. However, it was subject to systematic differences caused by the misrepresentation of the topography, requiring a bias correction prior to its use at high elevations^131^. CHIRTS is a merged product of station-based and reanalysis data. In its construction, it prioritised the estimation of Tmax rather than Tmin^9^, possibly explaining the significant differences with the latter variable. Considering the trends, there was a clear warming signal^5,38^, with larger magnitudes and spatially more homogeneously for Tmax than for Tmin^42^. CHIRTS and TerraClimate showed largest differences in temporal and spatial trends, leading to large unphysical trends due to unhomogenized or missing station data. This is an issue that should be fixed by using homogenisation algorithms.

#### Coastal fog effect on air temperature

In order to assess PISCOt v1.2 at a daily time step, we provide an analysis of the effect of coastal fog on modulating the daily mean air temperature. Coastal fog frequently occurs along the Peruvian coast and low Andean foothills. This phenomenon is especially persistent during austral winter (June-September), although it can occasionally appear throughout the year^132–134^. The occurrence of fog is often produced by the particular thermal inversion layer situation with cool lower air masses due to the south-north flowing Humboldt Current. The frequency of coastal fog increases gradually to the south, causing a marked diurnal cooling in the influenced coastal-Andean areas^134^.

We exemplified two situations with two variables: surface reflectance (Sref) from the MODIS terra satellite (MOD09GA version 6.1, band 1)^135^, which relates to the amount of cloud cover and the mean air temperature (Tmean: mean of Tmax and Tmin). This was performed during a coastal fog-covered (2007/08/25) and cloud-free (2006/08/24) day in northern Peru, including the Pacific Coast and Andean slopes^134^.

**Fig. 12:**
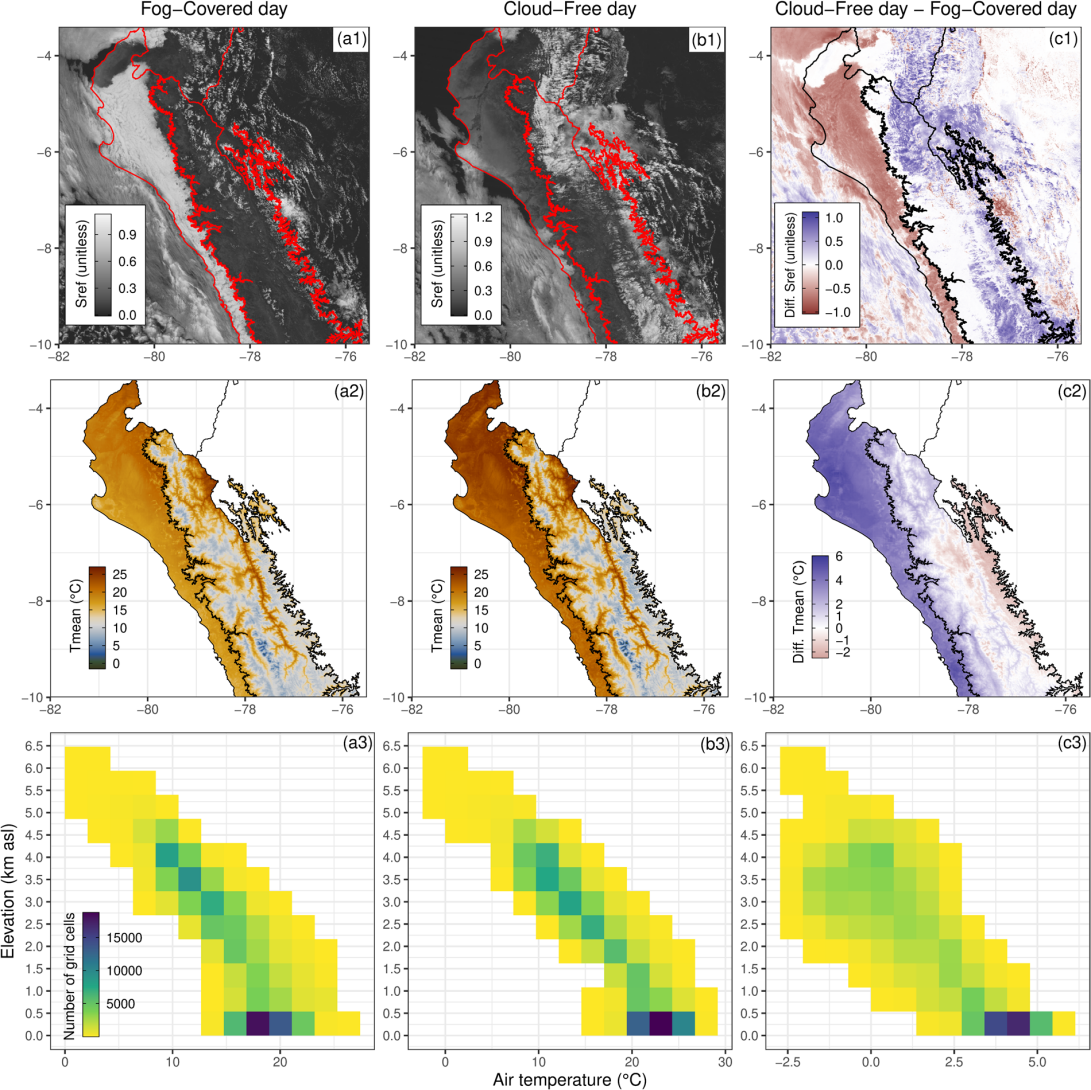
Examples of surface reflectance (Sref, MODIS-Terra) and mean air temperature (Tmean: mean of Tmax and Tmin) for two conditions of coastal fog-covered (a1 and a2) and cloud-free (b1 and b2) day for the northern regions of Peru (Pacific Coast and Andes). In addition, the difference between Sref and Tmean of both days is shown (c1 and c2; cloud-free day - fog-covered day). The vertical distribution of Tmean for both situations is shown in the lower panels as counts per air temperature values and elevation (a3, b3, and c3, respectively). c3 shows the number of grid cells with Tmean differences between −2.5 to 5.0 °C by elevation. Red (a1 and b1) and black (c1, a3, b3, and c3) lines represent the three main climate regions in Peru (Fig. 3).

Figure 12 shows the spatial variability of Sref and Tmean during the two situations and its spatial difference; in addition, the vertical distribution of Tmean with elevation was included. For the Pacific Coast area, Sref values were higher (more reflectance) on the fog-covered day than on the cloud-free day, reaching a contrast of up to −1 (Fig. 12a1,b1,c1). The negative differences in Sref revealed very well the spatial configuration of the fog. When inspecting Tmean, lower values were found on the fog-covered day compared to the cloud-free day, leading to differences of up to 2–6 °C; outlining certainly the Pacific Coast area (Figure 12a2,b2,c3). In this sense, there was a clear spatial contrast in both variables in the two situations: the higher the Sref values (more cloud cover), the lower the Tmean.

From a vertical perspective (Figure 12a3,b3,c3), it was also confirmed that there was a contrast on both days in low-elevation areas where the fog was located. We found that the higher Tmean differences were mostly present for grid cells below 500 masl. There are, however, also positive differences above 500 masl, but for much fewer grid cells. This high contrast in the number of grid cells determined the presence of fog on low levels. Interestingly, the value of 500 masl is close to the height of the thermal inversion layer of 400 masl identified in a previous study^134^. Furthermore, we found negative Tmean differences between both situations above 2500 masl (Figure 12c3), which can also be attributed to the presence of clouds at higher elevations on the cloud-free day rather than on the fog-covered day (Sref difference is positive in those areas).

These results suggest that PISCOt v1.2 is able to identify the effect of coastal fog on air temperature. Nevertheless, more in-depth analysis is required for a better understanding of this phenomenon.

## Usage Notes

The PISCOt v1.2 database is a valuable dataset for different applications in Peru as it allows for high-resolution analyses linked to e.g. climate change, health, hydrology, ecosystem assessments, and other fields for research and practitioners. PISCOt v1.2 supports the generation of new findings urgently required for more robust local decision-making in the scientific and political communities, especially in a context of data scarcity and high uncertainties in the region.

The new PISCOt v1.2 product has improved compared to the earlier version 1.1 in several key aspects: more assimilated time series, better consistency of station data pre-processing (quality control, gap-filling, and homogenisation), use of updated freely available auxiliary predictors, higher spatial resolution, a tidier and revised calculation sequence, and improved version control. Therefore, the development of PISCOt v1.2 is more consistent, traceable, and reproducible compared to other previously established gridded products in Peru.

PISCOt v1.2 adequately characterises the spatiotemporal variability of air temperature in average and extreme values using indicators. However, within the scope of this study only three indices were used. Future assessments therefore need to focus on more indicators of climate extremes not assessed in this study.

As the region is topographically complex, including steep climatic gradients, and is characterized by a low density and uneven distribution of weather stations, inherent limitations in spatial interpolation are expected, mainly at high elevations (between 1000 and 2000 masl, and >3500 masl). It is therefore recommended to use PISCOt v1.2 along with other gridded multi-source products which would allow for a better characterisation of the associated uncertainties in air temperature. More importantly, when aiming the evaluation of temporal trends on Tmin. A poor trend validation was found for Tmin that could lead to local erroneous climatic evaluation, in some cases with the opposite sign.

Furthermore, it is essential to clarify that matching weather stations with PISCOt v1.2 (and other products) is not recommended for assessing air temperature accuracy^136^. This is because such an analysis would favour products with interpolation algorithms that constrain the gridded data to precisely match weather station data. Likewise, if processes such as gap-filling, and homogeneity correction, among others, are applied to the observed data before spatial interpolation, the updated information would therefore no longer match the original data.

Finally, the gridded data of PISCOt v1.2 should only be used for continental areas. Due to the differences in LST values over water bodies compared to their surrounding terrestrial landscapes and the lack of observations over lakes, further validation is required to confirm the accuracy of spatial air temperature patterns over water^13^. Estimates over e.g. water bodies should therefore be masked out (i.e. be considered as empty grids).

### Supplementary information

SUPPLEMENTARY INFORMATION

## Data Availability

The construction of the gridded dataset PISCOt v1.2 was performed using the R (v3.6.3) and Python (v3.8.5) programming languages. The entire code used is freely available at figshare^137^ and GitHub (https://github.com/adrHuerta/PISCOt_v1-2) under the GNU General Public License v3.0.
