# Supplementary material for: High-resolution grids of daily air temperature for Peru - the new PISCOt v1.2 dataset
Source: Sci Data. 2023 Dec 1;10:847. doi: 10.1038/s41597-023-02777-w (PMC10692097; doi:10.1038/s41597-023-02777-w)
Supplement: Supplementary file 1 — SUPPLEMENTARY INFORMATION [file 41597_2023_2777_MOESM1_ESM.pdf]

Supplementary Information for "High-resolution grids of daily air temperature for Peru - the new PISCOt v1.2 dataset"

Adrian Huerta[1,2,9,*], Cesar Aybar[3,4], Noemi Imfeld[5,6], Kris Correa[1], Oscar Felipe-Obando[1], Pedro Rau[7], Fabian Drenkhan[8], and Waldo Lavado-Casimiro[1]

[1]Servicio Nacional de Meteorología e Hidrología (SENAMHI), Lima, Perú.
[2]Departamento de Física y Meteorología, Universidad Nacional Agraria La Molina (UNALM), Lima, Perú
[3]Image Processing Laboratory, University of Valencia, 46980, Valencia, Spain
[4]High Mountain Ecosystem Research Group, National University of San Marcos, 15081, Lima, Peru
[5]Institute of Geography, University of Bern, Bern, Switzerland
[6]Oeschger Centre for Climate Change Research, University of Bern, Bern, Switzerland
[7]Centro de Investigación y Tecnología del Agua (CITA), Departamento de Ingeniería Ambiental, Universidad de Ingeniería y Tecnología (UTEC), Lima, Perú
[8]Geography and the Environment, Department of Humanities, Pontificia Universidad Católica del Perú, Lima, Peru
[9]Present address: Institute of Geography and Oeschger Centre for Climate Change Research, University of Bern, Bern, Switzerland
[*]corresponding author(s): Adrian Huerta (adrhuerta@gmail.com)

Supplementary Figures

- Supplementary Figure 1. Number of observations for raw data and after quality control (QC): daily air maximum (Tmax, red) and minimum (Tmin, blue) temperature.

- Supplementary Figure 2. Number of deleted data by each quality control (QC) step for daily air maximum (Tmax, red) and minimum (Tmin, blue) temperature.

- Supplementary Figure 3. Boxplot of the number of available neighbouring weather stations at different distance and elevation levels (a). Daily mean correlation at different distance and elevation levels for air maximum (Tmax) and minimum (Tmin) temperature (b).

- Supplementary Figure 4. Example of daily air maximum (Tmax) and minimum (Tmin) temperature series with measurement precision inconsistencies: time series from the weather station (Observed) and PISCOt v1.2 (nearest Grid). Stations (a) PUCALA (longitude: -79.60°; latitude: -6.75°; elevation: 85 masl, region: Lambayeque) and (b) COLQUEPATA (longitude: -71.67°; latitude: -13.36°; elevation: 3696 masl, region: Cusco).

- Supplementary Figure 5. Annual Sen slope (1983-2013) of temperature indices (mean Tmax (MTmax), mean Tmin (MTmin), and frost days (FD)) for PISCOt v1.2 and gridded products (PISCOt v1.1, VS2018, TerraClimate, CHIRTS, and ERA5-Land) over southern Andes of Peru. Black lines represent three main regions: Pacific Coast, Andes, and Amazon; Lake Titicaca is shown as a lightblue filled area.

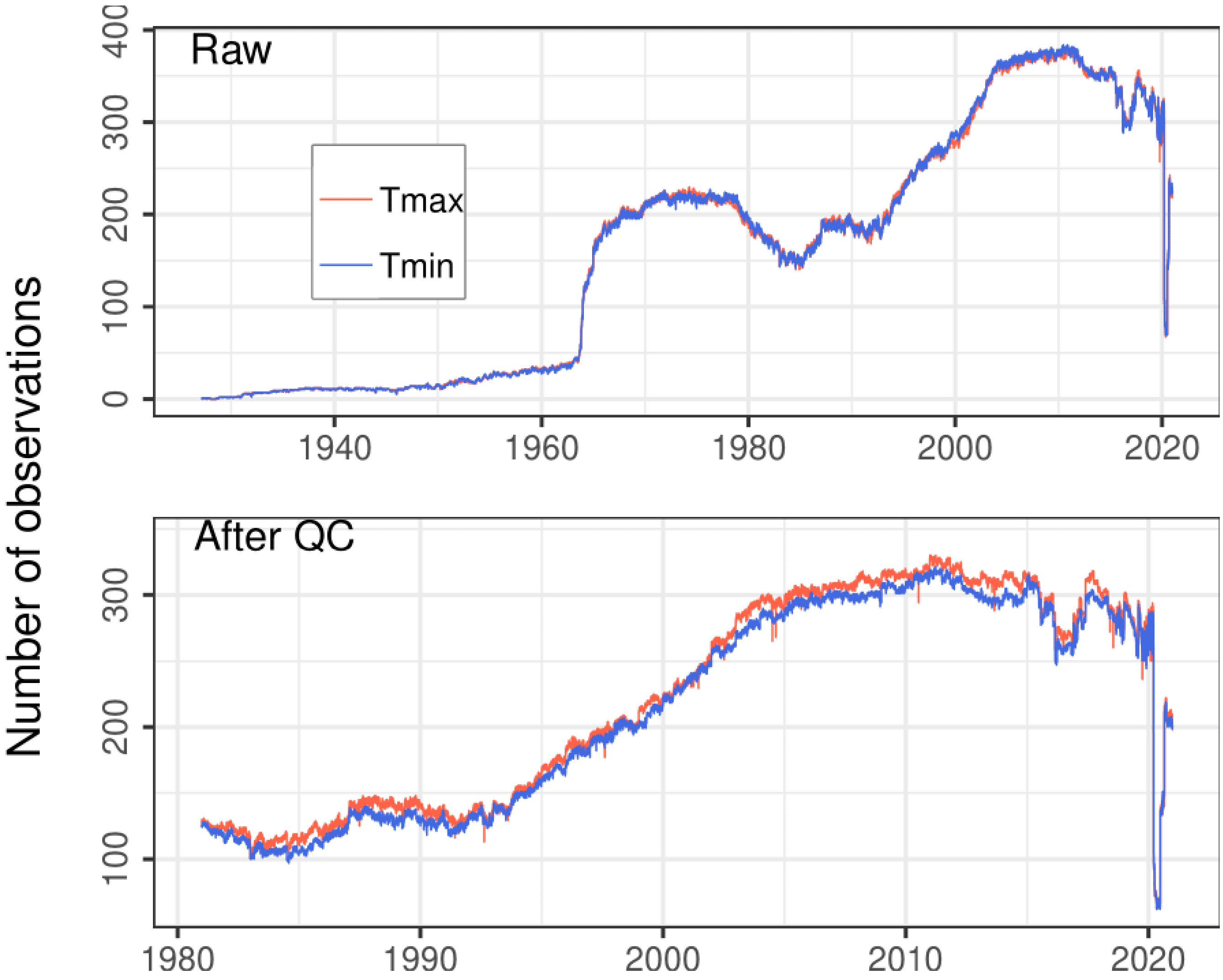

Supplementary Figure 1. Number of observations for raw data and after quality control (QC): daily air maximum (Tmax, red) and minimum (Tmin, blue) temperature.

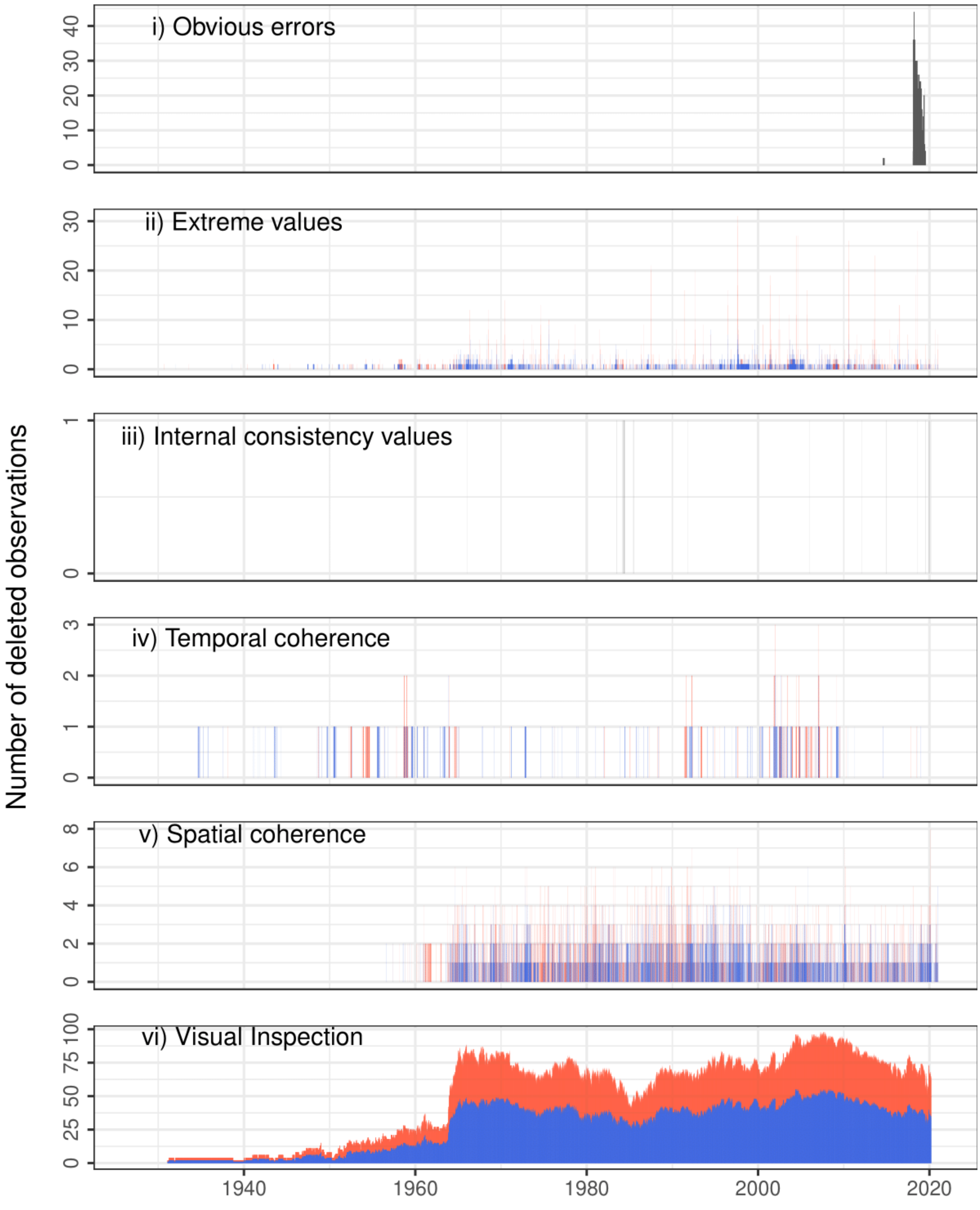

Supplementary Figure 2. Number of deleted data by each quality control (QC) step for daily air maximum (Tmax, red) and minimum (Tmin, blue) temperature.

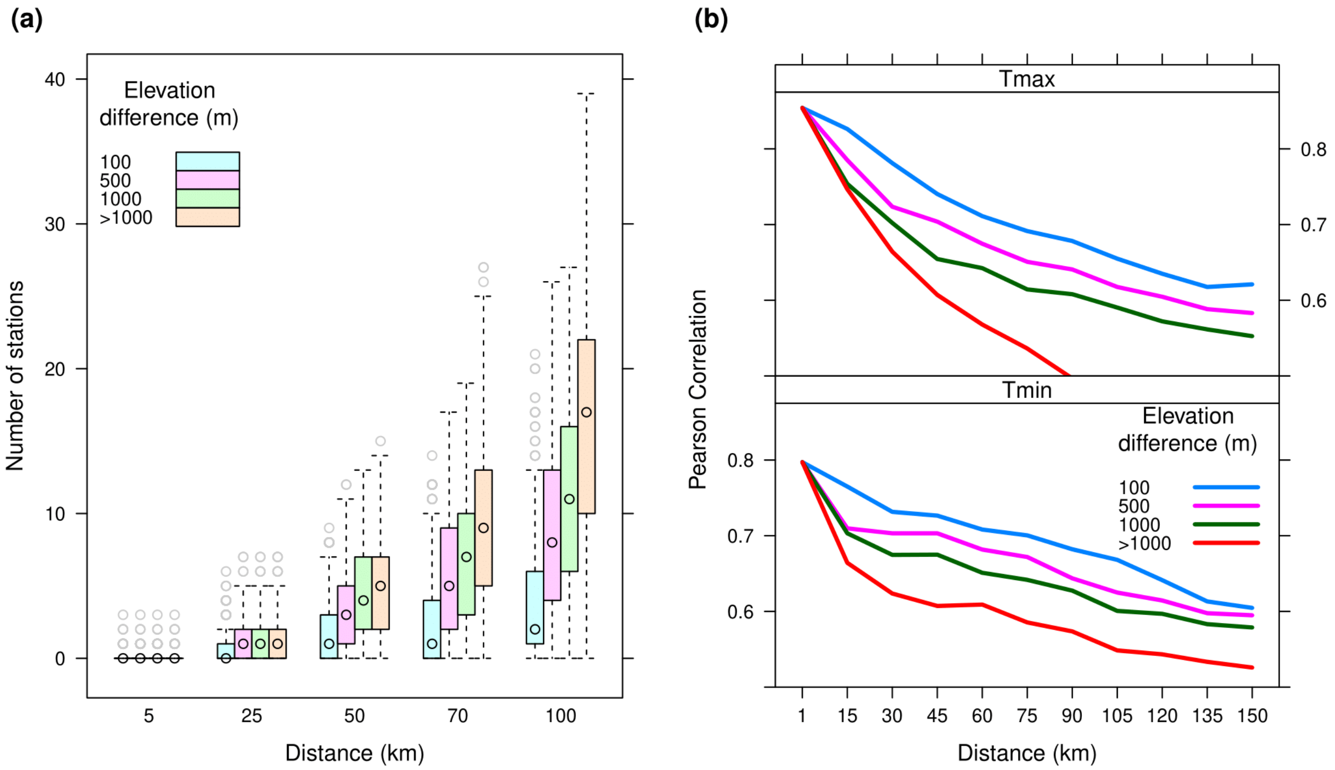

Supplementary Figure 3. Boxplot of the number of available neighbouring weather stations at different distance and elevation levels (a). Daily mean correlation at different distance and elevation levels for air maximum (Tmax) and minimum (Tmin) temperature (b).

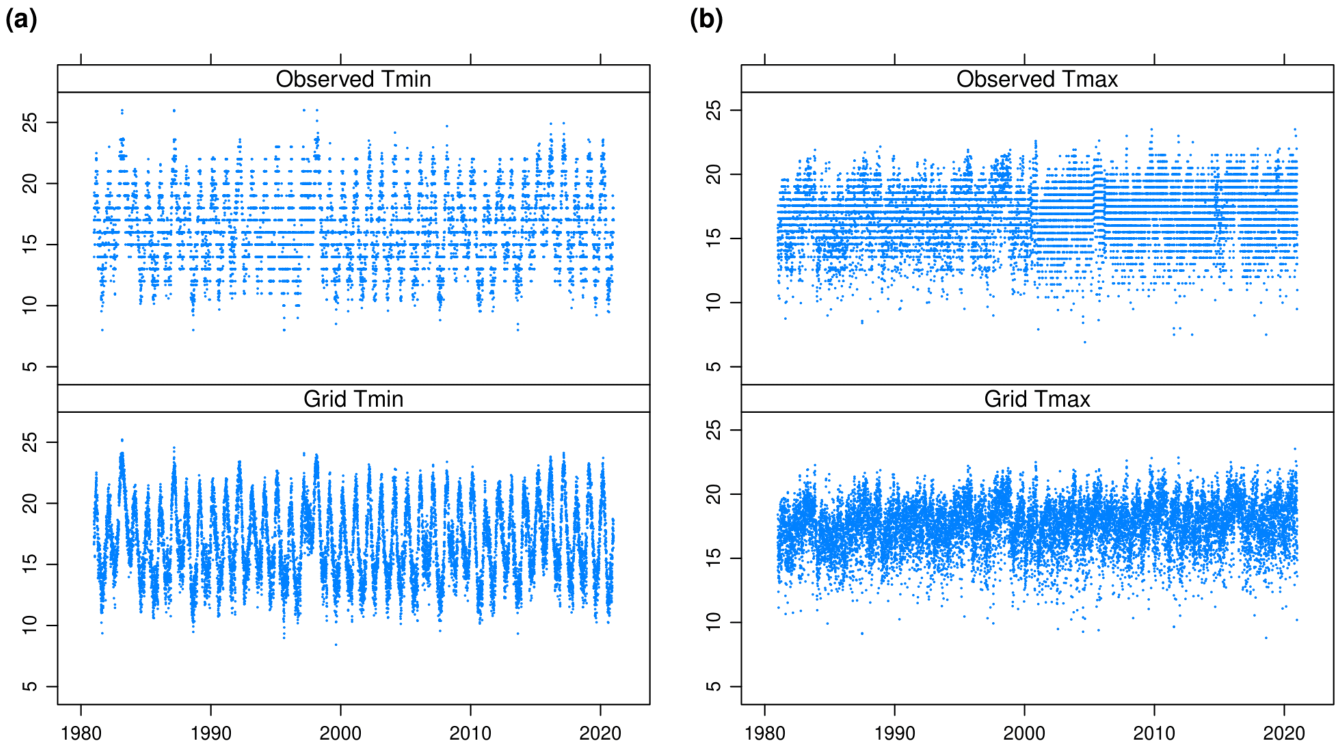

Supplementary Figure 4. Example of daily air maximum (Tmax) and minimum (Tmin) temperature series with measurement precision inconsistencies: time series from the weather station (Observed) and PISCOt v1.2 (nearest Grid). Stations (a) PUCALA (longitude: -79.60°; latitude: -6.75°; elevation: 85 masl, region: Lambayeque) and (b) COLQUEPATA (longitude: -71.67°; latitude: -13.36°; elevation: 3696 masl, region: Cusco).

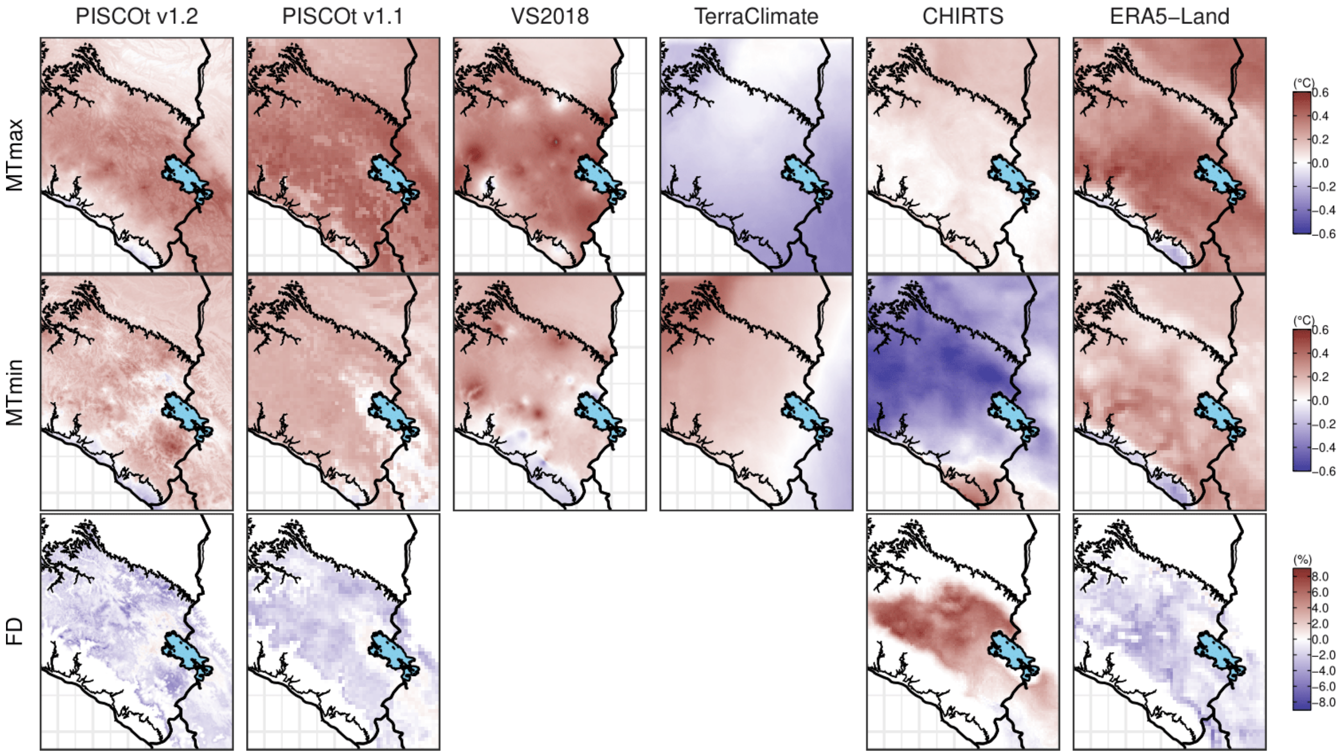

Supplementary Figure 5. Annual Sen slope (1983-2013) of temperature indices (mean Tmax (MTmax), mean Tmin (MTmin), and frost days (FD)) for PISCOt v1.2 and gridded products (PISCOt v1.1, VS2018, TerraClimate, CHIRTS, and ERA5-Land) over southern Andes of Peru. Black lines represent three main regions: Pacific Coast, Andes, and Amazon; Lake Titicaca is shown as a lightblue filled area.
